# *Clostridioides difficile* co-infection worsens prognosis in inflammatory bowel disease in patients with cytomegalovirus colitis

**DOI:** 10.1007/s00384-025-04954-2

**Published:** 2025-07-23

**Authors:** Ching-Reigh Hsieh, Chyi-Liang Chen, Chia-Jung Kuo, Ren-Chin Wu, Pai-Jui Yeh, Chien-Ming Chen, Cheng-Tang Chiu, Cheng-Hsun Chiu, Ming-Yao Su, Ming-Ling Chang, Yuan-Ming Yeh, Yu-Bin Pan, Puo-Hsien Le

**Affiliations:** 1 Department of Gastroenterology and Hepatology, Chang Gung Memorial Hospital, Guei-Shan District, 5, Fu-Hsin Street, Taoyuan City, Linkou, 33305 Taiwan; 2 Molecular Infectious Disease Research Center, Chang Gung Memorial Hospital, Taoyuan, Linkou, Taiwan; 3 Chang Gung Inflammatory Bowel Disease Center, Taoyuan, Linkou, Taiwan; 4 Chang Gung Microbiota Therapy Center, Taoyuan, Taiwan; 5 Taiwan Association for the Study of Intestinal Diseases (TASID), Taoyuan, Taiwan; 6 Department of Anatomic Pathology, Chang Gung Memorial Hospital, Taoyuan, Linkou, Taiwan; 7 Division of Pediatric Gastroenterology, Department of Pediatrics, Chang Gung Memorial Hospital, Taoyuan, Linkou, Taiwan; 8 Department of Medical Imaging and Interventions, Chang Gung Memorial Hospital, Taoyuan, Linkou, Taiwan; 9 Genomic Medicine Core Laboratory, Chang Gung Memorial Hospital, Taoyuan, Linkou, Taiwan; 10 Division of Pediatric Infectious Diseases, Department of Pediatrics, Chang Gung Memorial Hospital, Taoyuan, Linkou, Taiwan; 11 Division of Gastroenterology and Hepatology, Department of Internal Medicine, New Taipei Municipal Tucheng Hospital, Tucheng, New Taipei City, Taiwan; 12 Biostatistical Section, Clinical Trial Center, Chang Gung Memorial Hospital, Taoyuan, Linkou, Taiwan

**Keywords:** Cytomegalovirus, *Clostridioides difficile*, Inflammatory bowel disease, Opportunistic infection, Clinical remission

## Abstract

**Background:**

Cytomegalovirus (CMV) colitis and *Clostridioides difficile* infection (CDI) are both linked to disease exacerbation and poor prognosis in patients with inflammatory bowel disease (IBD). Nonetheless, the effect of co-infection on clinical outcomes in individuals with IBD remains underexplored. This retrospective study was designed to assess the clinical outcomes and determine predictors of co-infection with CMV and CDI in individuals with IBD.

**Methods:**

This analysis involved hospitalized patients with IBD and confirmed CMV colitis (based on intestinal CMV immunohistochemical staining) and *Clostridioides difficile* toxin A/B test results, collected at the Linkou branch of Chang Gung Memorial Hospital between January 2001 and September 2023. The individuals in the study cohort were divided into two categories: those with CMV infection alone and those with CMV/CDI co-infection. Clinical manifestations, outcomes, and independent predictors of co-infection were assessed between the two groups.

**Results:**

Overall, 53 IBD inpatients were enrolled in this study, with 37 assigned to the CMV group and 16 to the CMV/CDI co-infection group. The co-infection group experienced significantly more diarrhea (54.1% vs. 93.8%, *p* = 0.005) and abdominal pain (54.1% vs. 87.5%, *p* = 0.020) compared to the CMV group. Hospitalization duration (1 vs. 2.5 admissions, *p* = 0.005) and CMV recurrence (0 vs. 1 recurrences, *p* < 0.001) were higher in the co-infection group. Additionally, co-infection prolonged the time to clinical (1 vs. 5 months, *p* < 0.001), steroid-free (4 vs. 10 months, *p* = 0.001), endoscopic (8.3 vs. 17.5 months, *p* = 0.011), and histological remission (11 vs. 18 months, *p* = 0.021) compared to CMV infection alone. The cumulative incidence of clinical, steroid-free, endoscopic, and histological remission showed a delayed course in the co-infection group. Multivariable analysis revealed that biologic therapy was an independent predictor for CMV/CDI co-infection (OR 13.33, 95% CI 1.52–117.15, *p* = 0.02).

**Conclusion:**

Co-infection of CMV and CDI among individuals with IBD results in more frequent hospitalizations, higher CMV recurrence rates, and prolonged disease remission compared to CMV colitis alone. The administration of biologic therapy increases the risk of co-infection, emphasizing the importance of careful management in this patient population.

## Introduction

Inflammatory bowel disease (IBD), which includes ulcerative colitis (UC) and Crohn’s disease (CD), manifests as alternating periods of a relapsing–remitting pattern, with periods of symptom remission interrupted by disease flare-ups [1]. Distinguishing between idiopathic IBD flare-ups and opportunistic infections during periods of active disease is crucial due to the differing treatment strategies required. *Clostridioides difficile* and cytomegalovirus (CMV) are common opportunistic infections among IBD patients [2].

*Clostridioides difficile* is a spore-forming anaerobic bacterium widely recognized as a primary contributor to antibiotic-associated diarrhea [3, 4] and is a main trigger of healthcare-associated infections [4, 5]. Research has shown a rising global burden of *Clostridioides difficile* infection (CDI), with annual incidence rates ranging from 1.1 to 631.8 per 100,000 population [4, 6, 7]. Early research has demonstrated that the prevalence of CDI among hospitalized patients with UC ranged from 3.7 to 4.2% [10, 11], while more recent studies have shown an increasing trend, with rates ranging from 6 to 9% [12, 13]. Accumulating evidence also highlights the severity of CDI, its associated mortality [6, 9], and the significant global financial burden it imposes [8, 9]. Among individuals with CDI, those with concurrent IBD warrant particular attention. Patients with IBD who have concurrent CDI tend to have prolonged hospital stays [1], escalation in IBD therapy [2], and higher incidence of colectomy rate and mortality [2, 5, 15]. The risk factors of CDI among IBD patients included chronic exposure to antibiotics, steroids, and immunomodulators [11, 16]. Additionally, IBD is widely recognized as a significant and independent risk factor for the development of CDI [2] primarily due to the presence of mucosal defect and gut dysbiosis [6, 17]. Compared to the general population, the incidence of CDI is estimated to be nearly three times higher in patients with IBD [11].

Similarly, CMV, a member of the Herpesviridae family, is a significant opportunistic pathogen in hospitalized IBD patients [2, 18]. Individuals with IBD exhibit a substantially higher rate of CMV colitis incidence, particularly those with UC, compared to non-IBD patients [19]. Reported prevalence rates range from 10 to 30% in cases of steroid-refractory acute severe colitis [20]. CMV colitis in IBD patients has been correlated with serious complications, such as toxic megacolon, a higher risk of colectomy, IBD exacerbations, elevated inpatient mortality, extended hospital stays, and resistance to steroid therapies [21–23].

Despite the poor prognosis linked to both CMV colitis and CDI in individuals with IBD, there is a lack of research comparing the outcomes between CMV/CDI co-infection and CMV colitis alone. Furthermore, few investigations have explored how co-infection affects disease remission in individuals with IBD. To address these gaps, we investigate these issues in this study.

## Materials and methods

### Declarations

This study, focusing on the “Diagnosis, Treatment, and Prognosis of Inflammatory Bowel Disease,” was approved by the Institutional Review Board (IRB) of Chang Gung Medical Foundation (approval No. 202400030B0). Given its retrospective nature, individual patient consent for medical record review was waived by the IRB. The study complies with the ethical guidelines set by the 1975 Declaration of Helsinki.

### Patients

We examined medical records of IBD patients hospitalized at Linkou Chang Gung Memorial Hospital from January 2001 through September 2023. IBD patients hospitalized for acute flare-ups and complications management were included, with all subjects undergoing immunohistochemical (IHC) staining for CMV on colonic tissue samples. We excluded outpatients and individuals lacking CMV IHC results. CMV colitis was confirmed by detecting positive CMV IHC staining and observing tissue damage linked to CMV infection, which could manifest either in the presence or absence of viral inclusion bodies, as seen in hematoxylin and eosin-stained samples. IHC staining was performed with monoclonal antibodies targeting the CMV pp65 antigen (Leica Microsystems, Germany). CDI was diagnosed by identifying the presence of toxins A and B from *Clostridioides difficile* in stool samples. IBD individuals diagnosed with confirmed CMV colitis were classified into two groups: the co-infection group (CMV + CDI) and the CMV-only group, according to stool *C*. *difficile* toxin results.

### Management of CMV colitis and CDI

CMV colitis treatment was based on the European Crohn’s and Colitis Organization (ECCO) guidelines, which recommended an initial IV ganciclovir regimen of 5 mg/kg every 12 h that was maintained for 5 to 10 days, after which oral valganciclovir at a daily dose of 900 mg was prescribed for 2 to 3 weeks [2]. For the initial treatment of CDI, oral vancomycin 125 mg was given four times daily for 10 days, or alternatively, metronidazole was given at a dosage of 500 mg per oral route, taken three times daily for a duration of 10 days [26]. In cases of recurrent CDI, fidaxomicin or fecal microbiota transplantation was employed [26]. Clinical improvement, along with negative detection of stool toxins A and B, signified a complete cure. In this study, the term “biologics failure” denotes a situation where patients no longer respond effectively to one or multiple biologic treatments, while “biologics users” refers to those actively receiving such therapies.

### The definitions of remission of IBD

Clinical remission was defined according to the IOIBD consensus [26]. In UC, this included either achieving PRO2 (rectal bleeding score of 0 and stool frequency score of 0) or maintaining a partial Mayo score below 3, without any subscore surpassing 1. For CD, remission was considered achieved when PRO2 criteria were met (abdominal pain ≤ 1, stool frequency ≤ 3) or when the HBI score was under 5. Endoscopic remission in UC required a Mayo endoscopic subscore of 0 or a UCEIS score of ≤ 1, while in CD, it was defined as an SES-CD score below 3 or the absence of ulcers (SES-CD ulceration subscore = 0). Histological remission for UC corresponded to a Nancy index of less than 2.

### Data collection

We compiled an extensive dataset containing demographic and clinical details such as patient age, sex, body mass index (BMI), and comorbid conditions including CD, UC, diabetes, cirrhosis, coronary artery disease, end-stage renal disease, autoimmune disorders, and cancer. In addition, we collected data on the duration of IBD, baseline complications (such as fistulas, perforations, abscesses, colon cancer, strictures, and previous IBD surgeries), initial medications, the time point at which CMV colitis was diagnosed, and the presenting symptoms. Information regarding antiviral treatments and clinical outcomes—such as hospitalization frequency during follow-up, clinical and steroid-free remission status, steroid dosage alterations, CDAI, Mayo score, BMI, and complications (including fistula, perforation, stricture, colon cancer, abscess, IBD surgery, recurrence, and mortality)—were documented. Biochemical parameters including WBC, Hb, PLT, Cr, Bil, ALT, ALB, and CRP were also recorded. Regarding virological analysis, we included assessments for CMV pp65 antigenemia and CMV viremia. The Light-Mix® Kit for detecting human CMV (TIB Molbiol, Berlin, Germany) was utilized, with a cutoff set at Cp 35, targeting a 226 base pair region of the glycoprotein B gene and the COBAS® TaqMan® CMV Test (Roche Diagnostics, Branchburg, NJ, USA; cutoff: 150 copies/mL). Additionally, serological tests for both CMV and Epstein-Barr Virus (EBV) were performed.

### Statistical analyses

Continuous data are presented as medians with ranges, while categorical data are shown as frequencies and percentages. For the comparison of continuous variables, the Mann–Whitney *U* test was performed, and for categorical variables, *χ*^2^ and Fisher’s exact tests were applied. To assess the independent risk factors for CMV/CDI co-infection, logistic regression models were employed, initially incorporating variables with a *p*-value < 0.05 in univariate analysis. Variables that retained statistical significance (*p* < 0.05) were subsequently included in the multivariate analysis. Statistical significance was defined as *p* < 0.05, and findings are expressed as odds ratios (ORs) with 95% confidence intervals (CIs) and *p*-values. Statistical analyses were carried out with SPSS version 22.0 (IBM Corp.).

## Results

**Fig. 1:**
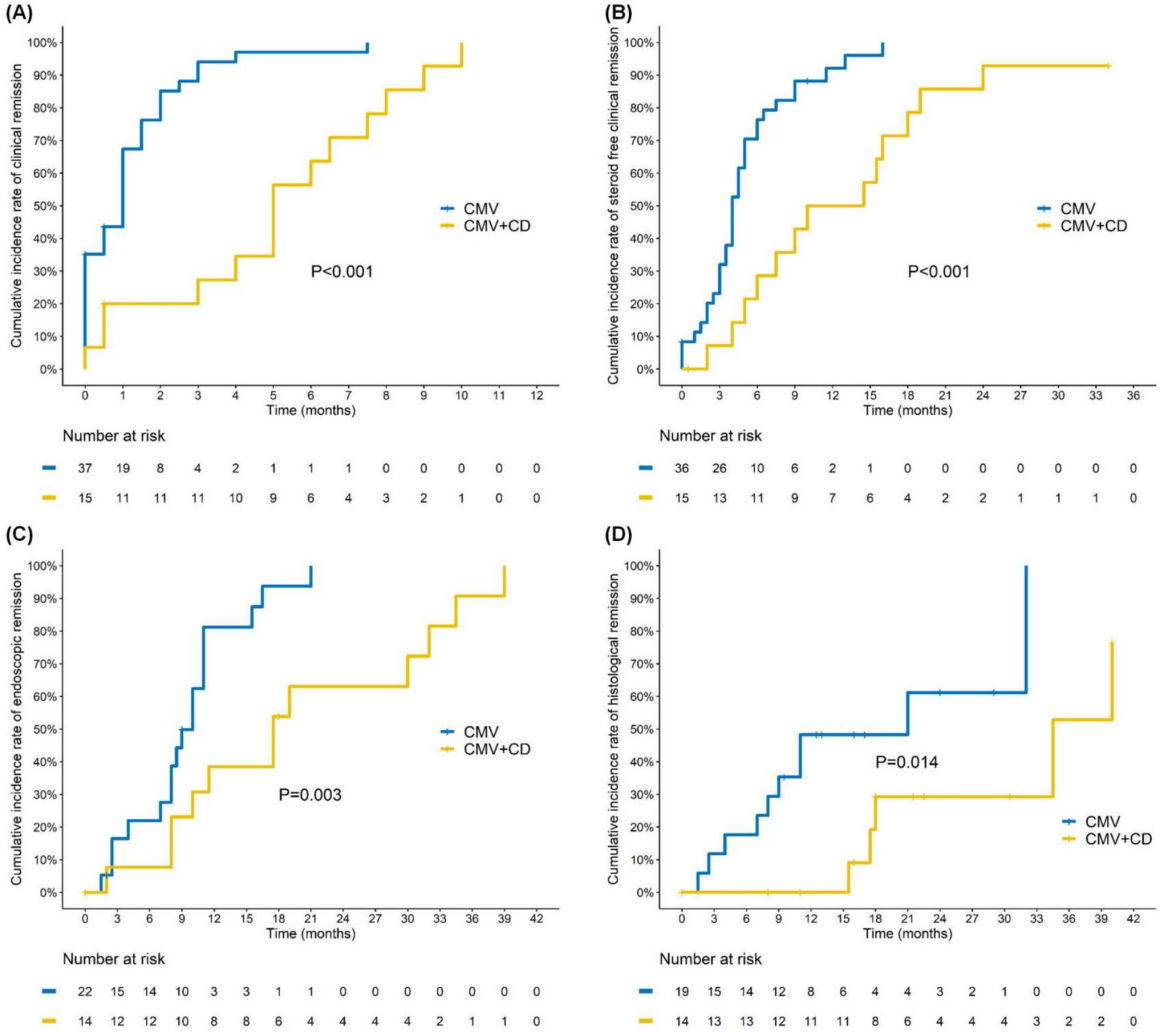
The cumulative incidence of **A** clinical remission, **B** steroid-free clinical remission, **C** endoscopic remission, and **D** histological remission in the CMV and CMV/CDI groups, as shown by Kaplan–Meier curves. Co-infection with CMV and *Clostridioides difficile* resulted in lower rates of clinical, steroid-free clinical, endoscopic, and histological remission compared to CMV infection alone

Initially, 136 patients were selected, but after excluding 83 individuals who were negative for CMV IHC or CDI, 53 hospitalized IBD patients were left for evaluation, with 37 patients in the CMV-only group and 16 in the group with both CMV and CDI. The patients had a median age of 45.3 years, with 71.7% being male. The follow-up period had a median length of 26.6 months. Age, gender, BMI, underlying health conditions, and baseline IBD complications did not differ significantly between the two groups. The co-infection group had significantly higher occurrences of diarrhea (93.8% vs. 54.1%, *p* = 0.005) and abdominal pain (87.5% vs. 54.1%, *p* = 0.02) compared to the CMV group (Table 1). However, the groups showed no significant discrepancies in their laboratory data (WBC, hemoglobin, creatinine, albumin, CRP) or in the administration of baseline drugs, including 5-ASA, oral prednisolone, and azathioprine. The CDI cohort was treated with metronidazole (*n* = 4), vancomycin (*n* = 4), or a combination of vancomycin and fecal microbiota transplantation (*n* = 6). All patients with CMV and CDI received intravenous ganciclovir for a minimum of 14 days. The co-infection group had a notably higher number of hospitalizations (2 vs. 1, *p* = 0.005) and CMV recurrences (1 vs. 0, *p* < 0.001) compared to those with CMV alone (Table 1). There were no significant differences between the groups regarding steroid dosage changes, CDAI, Mayo score adjustments, or overall IBD complications. The cohort was monitored for a median period of 34.8 months, with an interquartile range from 0.3 to 78.2 months. Patients in the co-infection group required significantly longer time to achieve specific clinical outcomes compared to those in the CMV group, including clinical remission (1 vs. 5 months, *p* < 0.001), steroid-free clinical remission (4 vs. 10 months, *p* = 0.001), endoscopic remission (8.3 vs. 17.5 months, *p* = 0.011), and histological remission (11 vs. 18 months, *p* = 0.021) (Table 1). Additionally, the cumulative incidence of clinical remission (*p* < 0.001), steroid-free remission (*p* < 0.001), endoscopic remission (*p* = 0.003), and histological remission (*p* = 0.014) was markedly higher in the co-infection cohort (Fig. 1). The multivariable analysis highlighted biologic therapy as an independent predictor of co-infection (OR = 13.33; 95% CI, 1.52–117.15; *p* = 0.02) (Table 2).

**Table 1:** Baseline characteristics of CMV/CDI and CMV groups in IBD patients

Characteristics	IBD (*n* = 53)	CMV + CDI (*n* = 16)	CMV (*n* = 37)	*p*
Age (years)	45.3 (3.4, 83.4)	46.4 (28.6, 70.4)	45.3 (3.4, 83.4)	0.543
Gender (male)	38 (71.7%)	11 (68.8%)	27 (73%)	0.751
BMI	21.1 (13.6, 30.2)	21 (13.6, 25.4)	21.5 (16.6, 30.2)	0.439
Baseline IBD characteristics				
Montreal classification for UC				
E1	2 (3.8%)	0 (0%)	2 (5.4%)	1.000
E2	19 (35.8%)	8 (50%)	11 (29.7%)	0.158
E3	20 (37.7%)	8 (50%)	12 (32.4%)	0.226
S1	6 (11.3%)	2 (12.5%)	4 (10.8%)	1.000
S2	30 (56.6%)	12 (75%)	18 (48.6%)	0.076
S3	5 (9.4%)	2 (12.5%)	3 (8.1%)	0.632
Montreal classification for CD				
B1	5 (9.4%)	1 (6.3%)	4 (10.8%)	1.000
B2	4 (7.5%)	0 (0%)	4 (10.8%)	0.303
B3	3 (5.7%)	0 (0%)	3 (8.1%)	0.545
L1	5 (9.4%)	0 (0%)	5 (13.5%)	0.307
L2	3 (5.7%)	1 (6.3%)	2 (5.4%)	1.000
L3	4 (7.5%)	0 (0%)	4 (10.8%)	0.303
L4	8 (15.1%)	1 (6.3%)	7 (18.9%)	0.410
CDAI	268.5 (197, 379)	316 (316, 316)	262 (197, 379)	0.311
SES-CD	9.5 (3, 22)	10 (10, 10)	10.3 (3, 22)	0.770
Mayo score	12 (7, 12)	12 (7, 12)	11 (7, 12)	0.362
Underlying diseases				
Hypertension	14 (26.4%)	6 (37.5%)	8 (21.6%)	0.311
Diabetes mellitus	9 (17%)	5 (31.3%)	4 (10.8%)	0.109
Coronary artery diseases	3 (5.7%)	1 (6.3%)	2 (5.4%)	1.000
Laboratory data				
WBC (1000/µL)	7.8 (3, 20.4)	9.5 (3, 12)	7.4 (4.2, 20.4)	0.500
Hemoglobin(g/dL)	11.2 (6.9, 15.9)	10.8 (7.4, 15)	11.5 (6.9, 15.9)	0.416
Creatinine	0.6 (0.2, 2.9)	0.6 (0.2, 1.2)	0.6 (0.3, 2.9)	0.436
Albumin (g/dL)	3 (1.4, 4.5)	2.4 (2, 4)	3.1 (1.4, 4.5)	0.199
CRP (mg/dL)	13.3 (0.2, 249)	19.1 (0.2, 74.7)	10.5 (0.3, 249)	0.984
Clinical presentation				
Bloody stool	41 (77.4%)	14 (87.5%)	27 (73%)	0.307
Diarrhea	35 (66%)	15 (93.8%)	20 (54.1%)	0.005*
Abdominal pain	34 (64.2%)	14 (87.5%)	20 (54.1%)	0.020*
Fever	11 (20.8%)	4 (25%)	7 (18.9%)	0.716
IBD medication				
5-ASA	11 (20.8%)	4 (25%)	7 (18.9%)	0.716
Oral prednisolone	11 (20.8%)	5 (31.3%)	6 (16.2%)	0.275
Azathiopurine Biological naiive Biological exposed Infliximab Adalimumab Vedolizumab Ustekinumab	8 (15.1%) 35 (66%) 18 (34%) 2 (3.8%) 4 (7.5%) 9 (17%) 2 (3.8%)	3 (18.8%) 10 (62.5%) 8 (50%) 1 (6.3%) 1 (6.3%) 6 (37.5%) 0 (0%)	5 (13.5%) 25 (67.6%) 10 (27%) 2 (5.4%) 3 (8.1%) 3 (8.1%) 2 (5.4%)	0.685 0.721 0.105 1.000 1.000 0.016 1.000
Baseline complications				
Stricture	7 (13.2%)	0 (0%)	7 (18.9%)	0.088
Perforation	5 (9.4%)	0 (0%)	5 (13.5%)	0.307
Abscess	8 (15.1%)	1 (6.3%)	7 (18.9%)	0.410
IBD related surgery	11 (20.8%)	1 (6.3%)	10 (27%)	0.141
Outcomes (overall)				
Steroid dose change	0 (− 60, 15)	0 (− 40, 15)	0 (− 60, 10)	0.440
Hospitalization times	2 (0, 27)	2.5 (1, 13)	1 (0, 27)	0.005*
CMV recurrent times	0 (0, 7)	1 (0, 7)	0 (0, 1)	0.003*
CDAI change	− 127.5 (− 322, 54)	− 170.6 (− 170.6, − 170.6)	− 100 (− 322, 54)	0.833
Mayo score change	− 7 (− 12, 1)	− 7 (− 11, 0)	− 7 (− 12, 1)	0.986
Clinical remission	49 (94.2%)	14 (93.3%)	35 (94.6%)	1.000
Duration	1 (0, 10)	5 (0, 10)	1 (0, 7.5)	< 0.001*
Steroid free clinical remission	46 (90.2%)	13 (86.7%)	33 (91.7%)	0.624
Duration	4.5 (0, 34)	10 (0.5, 34)	4 (0, 16)	0.001*
Endoscopic remission	29 (80.6%)	12 (85.7%)	17 (77.3%)	0.681
Duration	9.5 (0, 39)	17.5 (0, 39)	8.3 (0, 21)	0.011*
Histology remission	15 (45.5%)	5 (35.7%)	10 (52.6%)	0.335
Duration	15.5 (0, 40)	18 (0, 40)	11 (0, 32)	0.021*
IBD complications	23 (43.4%)	4 (25%)	19 (51.4%)	0.076
Stricture	12 (22.6%)	4 (25%)	8 (21.6%)	
Perforation	4 (7.5%)	1 (6.3%)	3 (8.1%)	
Abscess	5 (9.4%)	0 (0%)	5 (13.5%)	
Fistula	4 (7.5%)	1 (6.3%)	3 (8.1%)	
Surgery rate	17 (32%)	3 (18.8%)	14 (37.8%)	
Colon cancer	4 (7.5%)	1 (6.3%)	3 (8.1%)	
Death	3 (6.3%)	0 (0%)	3 (9.4%)	0.541
Follow-up duration (months)	26.6 (0.1, 123.1)	34.8 (0.3, 78.2)	23.9 (0.1, 123.1)	0.188

IBD complications included strictures, abscesses, fistulas, colon cancer, and IBD-related surgeries. *ALT* alanine aminotransferase, *5-ASA* 5-aminosalicylic acid, *BMI* body mass index, *CDAI* Crohn’s Disease Activity Index, *CI* confidence interval, *CMV* cytomegalovirus, *CRP* C-reactive protein, *IBD* inflammatory bowel disease, *WBC* white blood cell; *p < 0.05, calculated using the Mann–Whitney *U* tests for continuous variables while chi-square or Fisher’s exact test for categorical variables

**Table 2:** Independent risk factors for co-infection of *Clostridioides difficile* in IBD patients with CMV colitis

Characteristics	Univariable analysis			Multivariable analysis		
	OR	(95% CI)	*p*-value	OR	(95% CI)	*p*-value
Age (years)	1.013	(0.979–1.049)	0.453			
Gender (male)	0.815	(0.226–2.937)	0.754			
BW (kg)	0.990	(0.948–1.035)	0.669			
IBD duration	0.996	(0.985–1.006)	0.442			
BMI	0.893	(0.745–1.07)	0.219			
Underlying diseases						
Hypertension	2.175	(0.605–7.819)	0.234			
Diabetes mellitus	3.750	(0.853–16.495)	0.080			
CAD	1.167	(0.098–13.869)	0.903			
Laboratory data						
WBC (1000/μL)	0.986	(0.821–1.185)	0.879			
Segment	1.030	(0.984–1.079)	0.198			
Lymphocytes	0.979	(0.93–1.03)	0.411			
Hemoglobin (g/dL)	0.894	(0.689–1.161)	0.401			
Platelets	1.003	(0.999–1.007)	0.175			
CRP (mg/dL)	0.995	(0.98–1.011)	0.561			
Creatinine	0.398	(0.065–2.434)	0.318			
Albumin (g/dL)	0.494	(0.162–1.504)	0.215			
ALT	1.006	(0.978–1.036)	0.658			
Total bilirubin	0.023	(0–3.595)	0.143			
IBD Medication						
Biological failure	8.250	(1.98–34.381)	0.004	4.458	(0.958–20.744)	0.057*
Biologics user	19.687	(2.349–165.033)	0.006	13.327	(1.516–117.154)	0.020*
5-ASA	1.429	(0.353–5.788)	0.617			
Oral prednisolone	2.348	(0.596–9.258)	0.223			
Dosage (mg/day)	1.110	(0.944–1.305)	0.206			
Azathipurine	1.477	(0.307–7.098)	0.626			
Antibiotics	1.056	(0.327–3.411)	0.928			
Mesalazine enema	0.914	(0.158–5.293)	0.920			
Hydrocortisone enema	1.179	(0.193–7.193)	0.859			

IBD complications included strictures, abscesses, fistulas, colon cancer, and IBD-related surgeries. *ALT* alanine aminotransferase; *5-ASA* 5-aminosalicylic acid, *BMI* body mass index, *CDAI* Crohn’s Disease Activity Index, *CI* confidence interval, *CMV* cytomegalovirus, *CRP* C-reactive protein, *IBD* inflammatory bowel disease, *WBC* white blood cell, *OR* odds ratio; *p < 0.05, calculated using logistic regression analysis

## Discussion

The current therapeutic target in the management of IBD is to achieve steroid-free endoscopic remission. In the future, the focus will shift towards achieving histological remission in UC and transmural healing in CD, which represent deeper levels of disease control and improved long-term outcomes. Through a treat-to-target strategy, the ultimate goal is to reduce IBD-related complications and enhance patients’ quality of life [26, 27]. Our retrospective study aimed to fill this gap by comparing clinical remission (CR), steroid-free clinical remission (SFCR), endoscopic remission (ER), and histological remission (HR) in hospitalized IBD patients co-infected with CMV colitis and CDI versus those with CMV colitis alone.

Our findings revealed that biologic therapy significantly increases the likelihood of both CMV colitis and CDI among patients with IBD. Previous studies have demonstrated that immunosuppressive agents—including 5-ASA/6-MP, infliximab, and corticosteroids, especially when used in combination—along with old age, raise the likelihood of opportunistic infections in individuals with IBD [28]. Furthermore, IBD is independently linked to a higher risk of CDI [2]. The connection between biologic therapy and CDI risk was emphasized in a recent meta-analysis [29]. Additional cohort studies have identified biologics such as adalimumab and infliximab [30], corticosteroids [5, 30], and recent hospitalization within 1 month [5] correlated with an elevated risk of CDI. Interestingly, in our multivariate analysis, the administration of oral steroids, azathioprine, mesalazine enemas, or antibiotics did not appear to elevate the risk of co-infection among IBD patients, suggesting that biologic therapy plays a more prominent role in this regard.

Opportunistic infections not only trigger acute flare-ups but also lead to worse outcomes for IBD patients. Previous meta-analyses have indicated that CDI occurrence in IBD patients is connected to greater mortality rates in both the short and long term, along with an elevated risk of requiring colectomy [29]. In our study, when comparing patients with CMV colitis and CDI co-infection to those with CMV infection alone, no significant differences were observed in mortality or colectomy rates. Similarly, another study reported 1-year colectomy-free survival rates of 30% in the co-infection group and 57.1% in the CMV monoinfection group—a difference that did not reach statistical significance (*p* = 0.095) [1], further supporting the notion that co-infection does not significantly increase colectomy risk. Notably, our study is the first to demonstrate that co-infection is associated with delayed disease remission, more recurrent CMV cases (1 vs. 0), and more hospitalizations (2.5 vs. 1), further illustrating the additional burden that co-infection imposes on disease progression.

Although this study provides valuable insights, it is constrained by its retrospective design, the fact that it was conducted at a single center, and the small number of IBD patients with co-infection. Larger, multicenter prospective trials are needed to assess the effect of co-infection in IBD patients, particularly those undergoing biologic therapy. Future studies should also aim to elucidate the mechanisms underlying the increased co-infection risk in biologic users and evaluate the long-term outcomes of opportunistic infections in IBD management.

## Conclusion

IBD patients with concurrent CMV and CDI experience a higher rate of hospital admissions, higher CMV recurrence rates, and prolonged disease remission compared to CMV colitis alone. The administration of biologic agents contributes to an increased susceptibility to co-infection, highlighting the need for careful management in this patient population.

## Data Availability

No datasets were generated or analysed during the current study.
